# Multimeric forms of 4-1BBL as stimulators of T cells for adoptive immunotherapy

**DOI:** 10.1186/2051-1426-2-S3-P246

**Published:** 2014-11-06

**Authors:** Richard S Kornbluth, Victoria Snarsky, Geoffrey W Stone

**Affiliations:** 1 Multimeric Biotherapeutics, Inc., La Jolla, CA, USA; 2 University of Miami, Miller School of Medicine, Miami, FL, USA

## 

Members of the TNF SuperFamily of ligands (TNFSFs) have significant potential as immuno-oncology therapeutic agents. The TNFSFs are trimeric membrane proteins that can be cleaved into soluble single trimers. While the soluble single trimers can be easily prepared and studied, they have little or no activity in vivo. This deficiency is caused by the need to cluster their cognate receptors in the plane of the membrane in order to induce a supramolecular signaling complex on the cytoplasmic side of the plasma membrane. For the TNFSF ligands, this requires that they be used as many-trimer multimers that mimic the natural expression of many trimers on the surface of stimulating cells. To meet this need, we prepared fusion proteins comprised of the extracellular domains of TNFSF ligands joined to a natural protein that provides a multimerization scaffold. When Acrp30 (a natural serum protein) is used as a scaffold, the result is a 2-trimer TNFSF ligand product (MegaLigand™). When surfactant protein D (SPD) is used as a scaffold, the result is a 4-trimer TNFSF ligand product (UltraLigand™). Our published studies have described such multimeric forms of CD40L, OX40L, GITRL, CD27L/CD70, BAFF, RANKL, and TRAIL and shown that they are highly active in vitro and in vivo. As an extension of this work, 4-trimer forms of murine and human 4-1BBL (CD137L, TNFSF9) were constructed and expressed in CHO cells. As a co-stimulatory molecule, SPD-4-1BBL (Ultra4-1BBL™) promotes the proliferation of CD4+ and CD8+ T cells in vitro. Given the interest in 4-1BB (CD137) as a marker of therapeutically effective tumor-infiltrating lymphocytes (TILs), SPD-4-1BBL will be a useful growth factor for TIL manufacturing and T cell culturing in general.

